# Electrically-Tunable Blue Phase Liquid Crystal Microlens Array Based on a Photoconductive Film

**DOI:** 10.3390/polym12010065

**Published:** 2020-01-02

**Authors:** Bing-Yau Huang, Shuan-Yu Huang, Chia-Hsien Chuang, Chie-Tong Kuo

**Affiliations:** 1 Department of Physics, National Sun Yat-sen University, Kaohsiung 804, Taiwan; flyfishss31@gmail.com (B.-Y.H.); Kajon078350806@gmail.com (C.-H.C.); 2 Department of Optometry, Chung Shan Medical University, Taichung 402, Taiwan; syhuang@csmu.edu.tw; 3 Department of Ophthalmology, Chung Shan Medical University Hospital, Taichung 402, Taiwan; 4 Department of Optometry, Shu-Zen Junior College of Medicine and Management, Kaohsiung 821, Taiwan; 5 Innovation Incubation Center, Shu-Zen Junior College of Medicine and Management, Kaohsiung 821, Taiwan

**Keywords:** blue phase liquid crystals (BPLCs), microlens array, photo-induced conducting polymer, electrically switchable, polarization independence

## Abstract

This paper proposes an effective approach to fabricate a blue phase liquid crystal (BPLC) microlens array based on a photoconductive film. Owing to the characteristics of photo-induced conducting polymer polyvinylcarbazole (PVK), in which conductivity depends on the irradiation of UV light, a progressive mask resulting in the variation of conductivity is adopted to produce the gradient distribution of the electric field. The reorientations of liquid crystals according to the gradient distribution of the electric field induce the variation of the refractive index. Thus, the incident light experiences the gradient distribution of the refractive index and results in the focusing phenomenon. The study investigates the dependence of lens performance on UV exposure time, the focal length of the lens, and focusing intensities with various incident polarizations. The BPLC microlens array exhibits advantages such as electrically tunability, polarization independence, and fast response time.

## 1. Introduction

Liquid crystal (LC) lenses have extensive applications in many optical devices, such as optical communication [1], projection displays [2], signal processing to three-dimensional displays [3], data storage systems [4], and tunable photonic devices [1,5]. Gradient-index (GRIN) lens using LCs have attracted attention, possessing advantages such as planar surface structure and a smaller issue of LC alignment [5], simple fabrication process, and electrically induced non-uniform distribution of the LC directors [6,7,8]. However, because of the intrinsic uniaxial anisotropy of nematic LCs, the focusing properties of the LC lens depend strongly on the polarization of the incident light.

Blue phase liquid crystals (BPLCs) have recently become promising candidates for photonic applications [9,10,11,12,13,14,15,16,17,18,19]. BPLC lenses have been displayed experimentally with a fast response time [20], simple fabrication process [21], and the polarization-independent property [22,23]. To overcome the polarization dependence of conventional LC lens, polymer-stabilized blue-phase liquid crystals (PSBPLCs) and the Kerr effect have been considered as the better candidates [10,24]. Polarization-independent BPLC lenses have been proposed [11], but the fabrication process is complicated for the specific patterns of electrodes.

This paper presents a simple method to produce a BPLC microlens array with a photoconductive film polyvinylcarbazole (PVK). An exposure of UV light with gradient distribution of intensity results in the variation of conductivity on PVK, and thus the focusing effect and the tuning capability can be realized by the gradient distribution of the electric field. Through controlling the applied voltages on the sample, the focal length of the BPLC microlens array can be tuned. In addition, the proposed BPLC microlens exhibits independent polarization and fast response features.

## 2. Materials and Methods

The micro-array progressive mask was drawn by using AutoCAD and fabricated by Dabaodao Optoelectronics Co., Ltd. There are 16 × 16 circular patterns inside 1 cm^2^. The radius of each progressive circular pattern (R) is 200 μm. The distance (r) between circles is 200 μm. The light transmission of each circular pattern increases gradually from the center to the edge of the circle. The change in transmittance of the progressive circular pattern is with an interval width of 35 μm and 7 light-transmittance gradient distribution, at 0%, 7%, 10%, 25%, 50%, 53%, and 100%, from the center to the edge of the circular pattern.

**Figure 1:**
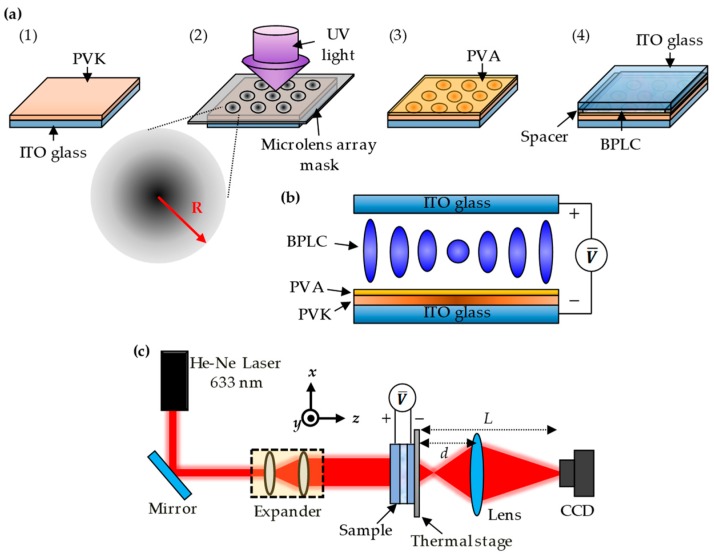
Schematic presentations of (**a**) the main production procedures of the sample: (**1**) PVK spin-coated; (**2**) UV exposure; (**3**) PVA layer assisted on the PVK film; (**4**) sample assembly; (**b**) a non-linear gradient of the LC orientation in the sample and thus the GRIN lens effect under an applied voltage; (**c**) the experimental setup for measuring the focal length of the BPLC microlens array.

Figure 1a presents the main production procedures of the sample as follows. (1) PVK spin-coated: The uniformly dissolved PVK solution (1.64 wt% in chlorobenzene) was spin-coated on the ITO substrate at 2600 rpm for 8 s. Next, the PVK-coated ITO substrate was placed in an oven and baked at 120 °C for 1 h to volatilize the chlorobenzene. (2) UV exposure: The PVK film was adhered to a progressive circular array mask and exposed by UV intensity of 260 mW/cm^2^ with various exposure times of 12, 14, and 16 h. The conductivity of PVK increases gradually from the center to the edge corresponding to the increase of light transmittance in each circular zone. (3) Polyvinyl alcohol (PVA, M_w_: 8.9 × 10^4^–9.8 × 10^4^) layer deposition: The exposed PVK substrate was immersed in the PVA solution (0.05 wt%) at 40 °C for 5 min and then baked at 120 °C for 20 min in order to prevent the PVK being dissolved in the BPLCs when the sample was heated above its clearing temperature [25]. (4) Sample assembly: The treated ITO substrate was assembled with another ITO-coated glass substrate with the spacer of 12 μm, and the BPLCs were filled into the assembled sample. The BPLCs were formed by mixing 60 wt% NLCs (HTW114200-100, *n*_e_ = 1.779 and *n*_o_ = 1.513 at 633 nm, from Fusol Material Co., Ltd.) and 40 wt% chiral dopant (S811, from Fusol Material Co., Ltd.).

The phase sequence of the BPLCs observed by the optical polarized microscope (OPM) is identified as ISO-42 °C-BPII-39.5 °C-BPI-36 °C-N*, where ISO and N* represent the isotropic phase and chiral nematic phase, respectively. When an external DC voltage was applied on an LC cell coated with a PVK layer, the effective voltage drop on the LC layer (*V*_LC_) in the steady-state regime is expressed as Equation (1) [26,27]:

(1) $$V_{\mathrm{LC}}\;=\;\frac{V}{{1+(d}_{\mathrm{PVK}}{\;\sigma }_{\mathrm{LC}})/{(d}_{\mathrm{LC}}{\;\sigma }_{\mathrm{PVK}})}$$

where *d*_PVK_ (*σ*_PVK_) and *d*_LC_ (*σ*_LC_) represent the thicknesses (conductivities) of the LC layer and the PVK layer, respectively. Equation (1) indicates that the voltage drop on the LC layer depends on the ratio of *σ*_LC_ to *σ*_PVK_. If the PVK film is conductive (*σ*_PVK_ > *σ*_LC_), then the largest external voltage can drop on the LC layer. If the PVK film is non-conductive (*σ*_PVK_ < *σ*_LC_), then only a fraction of the external voltage can drop on the LC layer. As the external DC voltage is applied to the cell, a non-linear gradient of an effective voltage drop on the LC layer can be obtained. This results in a non-linear gradient of LC orientation in the cell, which leads to an electrically tunable GRIN lens effect as shown in Figure 1b.

Figure 1c presents the experimental setup for the measurement of the focal length of the BPLC microlens array. The He-Ne laser was used as the detection beam, in which beam size was expanded to 1 cm of diameter through an expander and then passed through the sample. The sample was placed in a thermal stage of 38.5 °C. Based on Bragg′s diffraction, the wavelength of the reflection peak of BPLCs is about 492 nm at 38.5 °C. Since the BPLC microlens array had a short focal length, which was inconvenient to be recorded directly by the CCD camera. A convex lens was placed behind the focal plane of the sample, which allowed the image through the BPLC microlens array to easily focus on the CCD camera. In order to measure the focal length of the BPLC microlens array, the position of the image without applying an external voltage should be first obtained and then various voltages on the sample cell are applied to adjust the focal length of the microlens. The position of the lens was moved until the clear focusing image could be observed on the CCD camera. Herein, the focal length of the BPLC microlens array and convex lens are defined as *F* and *f*, respectively.

In Figure 1c the focal image formed by the BPLC microlens array can be regarded as an object corresponding to the convex lens, which once again focused on the CCD camera via a convex lens. According to the thin-lens theory, the equation of the convex lens can be written as:

(2) $$\frac{1}{f}=\frac{1}{p}+\frac{1}{q},$$

where *p* and *q* are respectively defined as the object and image distances, which are *d* − *F* and *L* − *d* from Figure 1c. Thus, Equation (2) can be rewritten as:

(3) $$\frac{1}{f}=\frac{1}{d-F}+\frac{1}{L-d}.$$

From Equation (3), the focal length (*F*) of the BPLC microlens array is calculated by Equation (4):

(4) $$F=\frac{{L(f-d)+d}^{2}}{d-L-f},$$

where *L* is the distance between the sample and the CCD camera, which was fixed at 27.5 cm; *f* was 5 cm here; *d* was a variable distance between the sample and the convex lens. To measure the polarization dependence of the BPLC microlens, a polarizer, and a quarter-wave plate were placed behind the beam expander to ensure that the detection beam was circularly polarized. Another polarizer plate was placed between the quarter-wave plate and the sample to adjust the incident polarization.

## 3. Results and Discussion

**Figure 2:**
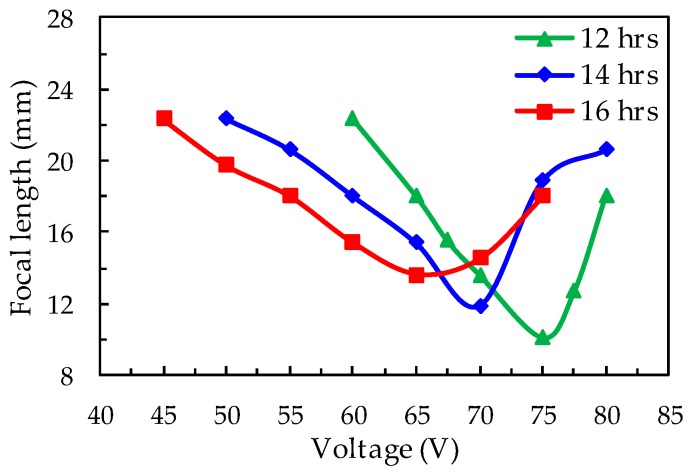
The dependence of the electrically controllable focal lengths of the BPLC microlens array with three different UV exposure time, 12 (green), 14 (blue), and 16 h (red).

Figure 2 presents the dependence of the electrically tunable focal length of the BPLC microlens array with three different UV exposure time, which is 12 h, 14 h, and 16 h, respectively. When no field is applied to the sample, the refractive index inside the sample is identical, and thus the focal length is infinity. When an electric field is applied to the sample, the lens-like behavior will occur due to the electrically induced GRIN effect. The minimum focal lengths were 10.11, 11.88, and 13.64 mm with the applied voltages of 75, 70, and 65 V, corresponding to the UV exposure time of 12 h, 14 h, and 16 h, respectively. We find that the minimum focal length rises under increasing UV exposure time. This phenomenon indicates that if PVK is over-exposed (16 h here), then conductivity will be saturated in the periphery area of the circular pattern, and it decreases the difference in the refractive indices in the central and periphery regions. From the GRIN lens theory, the focal length (*F*) is inversely proportional to the refractive index difference (Δn). Therefore, the focal length is relatively longer under the exposure time of 16 h. On the other hand, higher conductivity also leads to a smaller driving voltage. The driving voltage for the sample with a UV exposure time of 16 h to reach the minimum focal length is around 65 V, which is smaller compared to the other two samples with shorter UV exposure time. Considering the tunable range of the focal length and the driving voltage, the sample with the exposure time of 12 h was picked for the following experiments.

**Figure 3:**
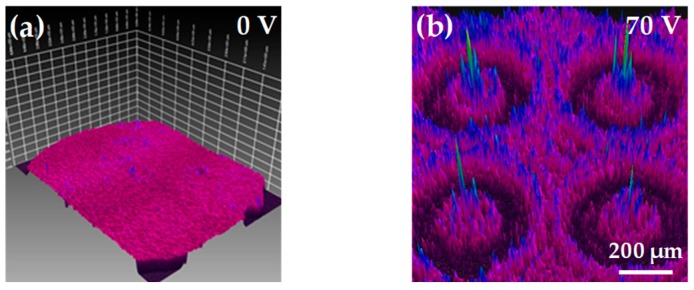
The 3D profiles of the beam intensity of the BPLC microlens array under the external voltage of (**a**) 0 V and (**b**) 70 V.

The optical properties in the focal plane of the BPLC microlens array could be captured and analyzed through the camera-based beam profiling system (known as BeamGage, from Ophir-Spiricon LLC, North Logan, UT, USA). Figure 3a presents the 3D profiles of the beam intensity of the BPLC microlens array under no external voltage. When a DC voltage of 70 V was applied on the BPLC microlens array, the 3D profiles of the beam intensity could be observed as shown in Figure 3b. The intensity of the detection beam is concentrated at the focal point of the BPLC microlens array. A focus performance could be achieved by applying the voltage in the BPLC microlens array.

**Figure 4:**
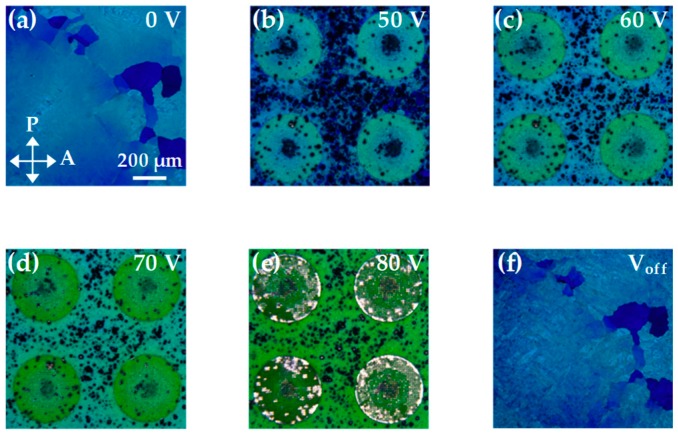
Microscopic images of the BPLC microlens array, which was placed between crossed polarizers, with the applied voltages of (**a**) 0 V; (**b**) 50 V; (**c**) 60 V; (**d**) 70 V; (**e**) 80 V; and (**f**) 0 V, respectively. The temperature was fixed at 38.5 °C.

Figure 4 presents the images of the BPLC microlens array, which was placed under crossed polarizers and operated with different voltages at 38.5 °C. When a reasonable voltage is applied to the sample, the BPLC microlens exhibits a color change due to the electrostriction effect [28]. At a voltage of 50 V, the effective electric field at the edge of the microlens is stronger than that at the center. Therefore, the refractive index of the BPLCs at the edge is modulated first and results in the optical focus. This phenomenon confirms that when the applied voltage is small, the tunability of the focal length will be mainly dominated by the peripheral phase change of the BPLCs. When the voltage gradually increases to 70 V, the minimum of the focal length can be obtained, because of the maximum phase difference between the center and the edge of the microlens. When the voltage exceeds 70 V, the BPLCs at the center of the microlens are also affected by the electrostriction effect under the strong electric field, so that the difference in the refractive indices (Δn) between the center and the periphery area decreases and results in a longer focal length, as shown in Figure 2. At a voltage of 80 V, the BPLCs start to become untwisted and undergo a phase change. At this stage, the difference in the refractive indices between the central and periphery regions decreases again, and thus the focal length increases. When the applied voltage is turned off (V_off_), the BPLCs returned to the initial state. This phenomenon implies that the electrical switchability of the BPLC microlens array is reliable.

**Figure 5:**
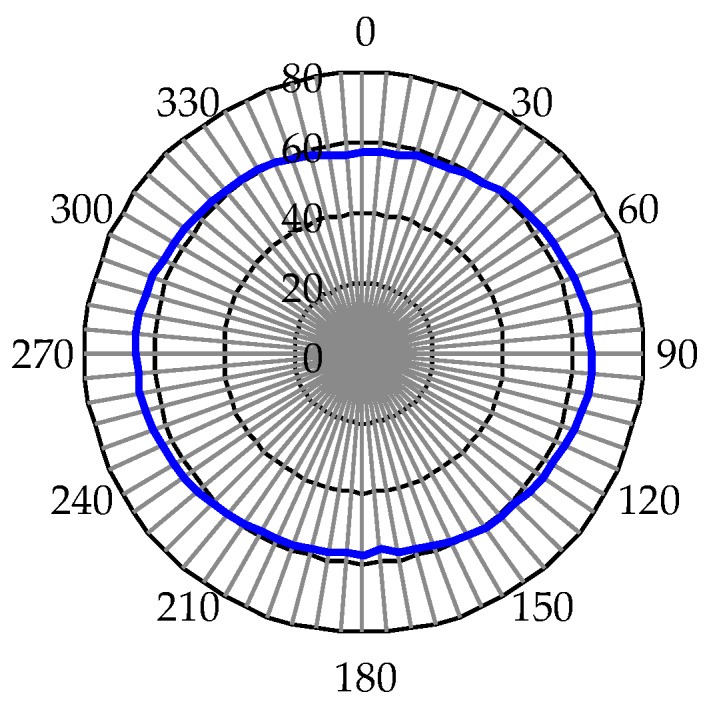
Variations of the incident polarization on the focusing intensities of the BPLC microlens array under the applied voltage of 70 V.

Figure 5 presents the intensities of a focus spot versus the incident polarization direction of the detection beam under the applied voltage of 70 V. The incident polarization is rotated from 0 to 360 degrees, and the intensities are recorded every five degrees. The focusing intensity approaches a value of ~61 at different incident polarization angles. This result implies that this BPLC microlens array possesses a promising polarization-independent characteristic.

**Figure 6:**
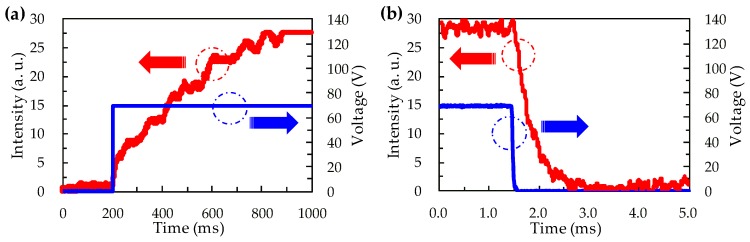
Response time of the BPLC microlens array with the voltage (**a**) switched on to 70 V, and (**b**) switched off from 70 V to 0 V.

Aside from the advantage of the polarization-independent property, the BPLC microlens array also exhibits a fast response time, which is in the order of milliseconds. The rise time (τ_rise_) and the decay time (τ_decay_) are measured as 508 ms and 940 μs, corresponding to the switching times from 0 to 70 V and from 70 to 0 V, respectively, as shown in Figure 6. The rise and decay times were defined as 10%–90% of the intensity change of the detection beam. The millisecond scale of rise time is attributed to the slower charging process of PVK [29]. However, both the rise and decay time of the BPLC microlens array are much smaller than those of the NLC microlens array driven by the PVK and DC voltage.

## 4. Conclusions

This paper has demonstrated an electrically tunable BPLC microlens array based on a photoconductive polymer layer, PVK. Progressive conductivity of the PVK layer is obtained by exposure to the UV light through a progressive circular array mask. As the external DC voltage is applied to the sample, a non-linear gradient of an effective voltage drop on the BPLC layer is obtained. This forms a non-linear gradient of LC orientation in the cell and results in an electrically tunable BPLC microlens array. The BPLC microlens array has the advantages of electrically-tunable focal length, polarization-independent, and fast response. However, several drawbacks have to be valued, such as the narrow temperature range of BPLCs, severe chromatic aberrations, and high operating voltage.

## References

[1] Ferstl M., Frisch A.M. (1996). Static and dynamic Fresnel zone lenses for optical interconnections. J. Mod. Opt..

[2] Davis A., Bush R.C., Harvey J.C., Foley M.F. (2001). P-95: Fresnel lenses in rear projection displays. SID Symp. Dig. Tech. Pap..

[3] Lu J.G., Sun X.F., Song Y., Shieh H.P.D. (2011). 2-D/3-D switchable display by Fresnel-type LC lens. J. Displ. Technol..

[4] Rastani K., Marrakchi A., Habiby S.F., Hubbard W.M., Gilchrist H., Nahory R.E. (1991). Binary phase Fresnel lenses for generation of two-dimensional beam arrays. Appl. Opt..

[5] McManamon P.F., Dorschner T.A., Corkum D.L., Friedman L.J., Hobbs D.S., Holz M., Liberman S., Nguyen H.Q., Resler D.P., Sharp R.C. (1996). Optical phased array technology. Proc. IEEE.

[6] Naumov A.F., Loktev M.Y., Guralnik I.R., Vdovin G.V. (1998). Liquid-crystal adaptive lenses with modal control. Opt. Lett..

[7] Kowel S.T., Cleverly D.S., Kornriech P.G. (1984). Focusing by electrical modulation of refraction in a liquid crystal cell. Appl. Opt..

[8] Ye M., Sato S. (2002). Optical properties of liquid crystal lens of any size. Jpn. J. Appl. Phys..

[9] Hong H.K., Jung S.M., Lee B.J., Shin H.H. (2009). Electric-field-driven LC lens for 3-D/2-D autostereoscopic display. J. Soc. Inf. Disp..

[10] Lin Y.H., Chen H.S., Lin H.C., Tsou Y.S., Hsu H.K., Li W.Y. (2010). Polarizer-free and fast response microlens arrays using polymer-stabilized blue phase liquid crystals. Appl. Phys. Lett..

[11] Li Y., Wu S.T. (2011). Polarization independent adaptive microlens with a blue-phase liquid crystal. Opt. Express.

[12] Chu F., Dou H., Li G.P., Song Y.L., Li L., Wang Q.H. (2018). A polarisation-independent blue-phase liquid crystal lens array using gradient electrodes. Liq. Cryst..

[13] Cui J.P., Fan H.X., Wang Q.H. (2017). A polarisation-independent blue-phase liquid crystal microlens using an optically hidden dielectric structure. Liq. Cryst..

[14] Yan J., Li Y., Wu S.T. (2011). High-efficiency and fast-response tunable phase grating using a blue phase liquid crystal. Opt. Lett..

[15] Yan J., Li Q., Hu K. (2013). Polarization independent blue phase liquid crystal gratings based on periodic polymer slices structure. J. Appl. Phys..

[16] He W., Pan G., Yang Z., Zhao D., Niu G., Huang W., Yuan X., Guo J., Cao H., Yang H. (2009). Wide blue phase range in a hydrogen-bonded self-assembled complex of chiral fluoro-substituted benzoic acid and pyridine derivative. Adv. Mater..

[17] Wang L., He W., Xiao X., Meng F., Zhang Y., Yang P., Wang L., Xiao J., Yang H., Lu Y. (2012). Hysteresis-free blue phase liquid-crystal-stabilized by ZnS nanoparticles. Small.

[18] Wang L., He W., Xiao X., Wang M., Wang M., Yang P. (2012). Low voltage and hysteresis-free blue phase liquid crystal dispersed by ferroelectric nanoparticles. J. Mater. Chem..

[19] Wang M., Zou C., Sun J., Zhang L., Wang L., Xiao J., Li F., Song P., Yang H. (2017). Asymmetric tunable photonic bandgaps in self organized 3d nanostructure of polymer stabilized blue phase I modulated by voltage polarity. Adv. Funct. Mater..

[20] Xu D., Peng F., Wu S.T. (2011). Polymer-stabilized blue phase liquid crystals. Opt. Mater. Express.

[21] Li Y., Liu Y., Li Q., Wu S.T. (2012). Polarization independent blue-phase liquid crystal cylindrical lens with a resistive film. Appl. Opt..

[22] Lin S.H., Huang L.S., Lin C.H., Kuo C.T. (2014). Polarization-independent and fast tunable microlens array based on blue phase liquid crystals. Opt. Express.

[23] Li Y., Huang S.J., Zhou P.C., Liu S.X., Lu J.G., Li X., Su Y.K. (2016). Polymer-Stabilized Blue Phase Liquid Crystals for Photonic Applications. Adv. Mater. Technol..

[24] Chen K.M., Gauza S., Xianyu H., Wu S.T. (2010). Submillisecond gray-level response time of a polymer-stabilized blue-phase liquid crystal. J. Displ. Technol..

[25] Chen Y.D., Fuh A.Y.G., Cheng K.T. (2012). Particular thermally induced phase separation of liquid crystal and poly(N-vinyl carbazole) films and its application. Opt. Express.

[26] Lo K.C., Wang J.D., Lee C.R., Mo T.S. (2007). Electrically controllable and polarization-independent Fresnel zone plate in a circularly symmetric hybrid-aligned liquid crystal film with a photoconductive polymer layer. Appl. Phys. Lett..

[27] Vladimirov F.L., Chaika A.N., Morichev I.E., Pletneva N.I., Naumov A.F., Loktev M.Y. (2000). Modulation characteristics of optically controllable transparencies based on a photoconductor–liquid-crystal structure. J. Opt. Technol..

[28] Chen C.W., Li C.C., Jau H.C., Yu L.C., Hong C.L., Guo D.Y., Wang C.T., Lin T.H. (2015). Electric field–driven shifting and expansion of photonic band gaps in 3D liquid photonic crystals. ACS Photonics.

[29] Dyadyusha A., Kaczmarek M., Slussarenko S. (2003). Dynamics and uniformity of reorientation in liquid crystal cells with PVK alignment layers. J. Electron. Liq. Cryst. Commun..

